# Correctional “Free Lunch”? Cost Neglect Increases Punishment in Prosecutors

**DOI:** 10.3389/fpsyg.2021.778293

**Published:** 2021-11-12

**Authors:** Eyal Aharoni, Heather M. Kleider-Offutt, Sarah F. Brosnan

**Affiliations:** ^1^Department of Psychology, Georgia State University, Atlanta, GA, United States; ^2^Department of Philosophy, Georgia State University, Atlanta, GA, United States; ^3^Neuroscience Institute, Georgia State University, Atlanta, GA, United States; ^4^Center for Behavioral Neuroscience, Atlanta, GA, United States

**Keywords:** prosecution, sentencing, punishment, decision making, cost-benefit analysis, cost discounting

## Abstract

Prosecutors can influence judges’ sentencing decisions by the sentencing recommendations they make—but prosecutors are insulated from the costs of those sentences, which critics have described as a correctional “free lunch.” In a nationally distributed survey experiment, we show that when a sample of (*n*=178) professional prosecutors were insulated from sentencing cost information, their prison sentence recommendations were nearly one-third lengthier than sentences rendered following exposure to direct cost information. Exposure to a fiscally equivalent benefit of incarceration did not impact sentencing recommendations, as predicted. This pattern suggests that prosecutors implicitly value incorporating sentencing costs but selectively neglect them unless they are made explicit. These findings highlight a likely but previously unrecognized contributor to mass incarceration and identify a potential way to remediate it.

## Introduction

Scientific attempts to understand the high rates of incarceration in the U.S. frequently implicate tough-on-crime policies like mandatory sentencing laws, the war on drugs, economic disparities, the privatization of prisons, and a long history of racial bias (see Travis et al., 2014). Less attention has been paid to the roles of prosecutors, who have wide discretion in deciding whether or not to file criminal charges, which charges are filed, and even what sentence will be considered (see Wise et al., 2009; Byers et al., 2012; Bushway et al., 2014; Robertson et al., 2019; Eck and Crabtree, 2020).

As adversaries to the defendant, prosecutors face a unique moral hazard: while they often stand to gain professionally by negotiating for harsh sentences, they are not required to answer for the costs of those sentences, which are paid by other levels of government. Thus, the professional benefits of the sentence are salient features of the sentencing choice architecture, but the financial costs of those sentences are diffused and therefore less salient (Misner, 1995; Pfaff, 2017; Miller, 2019).

These costs are not trivial. States pay an average of $33,000 per year in direct costs to incarcerate a typical offender (Mai and Subramanian, 2017), not including the many collateral (e.g., Kirk and Wakefield, 2018) or criminogenic consequences (e.g., Stemen, 2017) of incarceration, whose impact is most strongly felt by poor and minority communities (Bierschbach and Bibas, 2017). These state expenditures also entail opportunity costs that might be relevant to the public interest, such as the ability to fund policing, victim services, or offender services known to reduce the risk of reoffending. Yet, with rare exceptions (e.g., Colorado, Missouri), cost information is scarcely presented at sentencing, if not outright excluded from the sentencing process (see Nardulli, 1984; United States v. Park, 2014; Allyn, 2018).

Scholars have suggested that when prosecutors lack information about sentencing costs or incentives to consider them, they will pursue harsher sentencing strategies (Misner, 1995; Gold, 2011; Bierschbach and Bibas, 2017; Miller, 2019), which some critics have described as a “correctional free lunch” (Zimring and Hawkins, 1991, 211–215). This concern has fueled new policy efforts (e.g., California Bill AB 1474) to increase transparency in sentencing by disclosing sentencing cost estimates to prosecutors (Allyn, 2018; Alpert, 2021). Among professional judges, there is some evidence that increasing sentencing costs salience reduces the severity of their sentencing judgments (Rachlinski et al., 2013). However, it remains unknown whether prosecutors, who set the stage for judges, spontaneously take the financial costs of their sentencing recommendations into account, and whether increasing the salience of sentencing costs will affect their sentencing recommendations.

The present study represents the first systematic test of sentencing cost-benefit salience on sentencing judgments in a sample of professional prosecutors. We hypothesized (a) that sentencing recommendations would be lower when information about the costs of incarceration is present than when it is absent, but (b) sentencing recommendations would not differ when benefits information is present vs. absent because benefit information is already salient for prosecutors and therefore already incorporated into their sentencing judgments. Evidence that prosecutors’ sentencing recommendations are responsive to exposure to the costs of incarceration but not the benefits would suggest that they derive utility from cost information but selectively neglect to consider it without prompting. Such a finding would be important for policymakers tasked to manage tradeoffs between incarceration rates and other competing social services. It would also help legal practitioners and their electorates appraise and remediate the impact of selective information transparency on the criminal justice system and the communities that support it.

## Materials and Methods

### Participants

Two hundred fifty-four consenting prosecuting attorneys were recruited from the membership of one of three national, non-profit professional associations or one of five county-level district attorney’s offices in the United States (in CA, GA, MA, MO, and VA). District attorney’s offices with policies that require prosecutors to reference the costs of incarceration at sentencing were not included. However, eligible participants were free to redistribute the survey invitation to known prosecutors from other jurisdictions. To be eligible to participate, respondents reported having served as a prosecutor at any court in the United States for at least 6months. In accordance with our planned exclusion criteria, sixty-five participants were excluded for incomplete data, defined by failure to complete the primary dependent measures and/or a multiple-choice question assessing memory for the crime portrayed. Nine more were excluded for failing to recognize the monetary amount presented. Two more were excluded for submitting the survey in less than 2minutes. All of these exclusions were designed to remove participants who were likely unengaged in the task. The remaining sample of 178 participants reportedly was 56.7% female, 35.4% male (7.9% other or unspecified), with a mean age of 42.1years (*SD*=9.6). Mean years of experience as a prosecutor was 11.2 (*SD*=7.4). Thirty-five U.S. states were represented, including all four geographic regions (Northeast, South, Midwest, West). Multiple levels of government were represented, predominantly county (68.0%), followed by state (18.0%), city (6.2%), and other/prefer not to answer (7.9%). Self-reported political ideology was *M*=−0.83 (*SD*=1.35) on a 7-point bipolar scale ranging from (−3) “very liberal” to (+3) “very conservative.” Race and ethnicity were not collected.

### Design

Participants read a criminal case summary describing a fictitious defendant convicted of distribution of controlled substances. A drug trafficking crime was selected because drug offenses are highly represented in prison populations—e.g., 46% in federal prisons (Carson, 2020)—and yet public support for the prosecution of drug crimes has waned in recent years (Pew Charitable Trusts, 2018), making them a meaningful target for debate on policy reform. After reading the case summary, participants rendered punishment judgments following one of three experimentally controlled message types in a three-groups design: cost information present (cost condition), benefit information present (benefit condition), or no cost-benefit information present (unspecified control condition). The cost condition reported an estimated direct cost of $30,000 to $35,000 per year to incarcerate the offender (Rachlinski et al., 2013; Mai and Subramanian, 2017). The benefit condition reported a savings of the same amount ($30,000 to $35,000 per year) in the form of reduced crime. The benefit value was matched to the cost estimate for experimental control purposes. To help make the decision more concrete, both conditions also described a plausible opportunity cost (or benefit), namely that the funding could otherwise be (can instead be) spent on other correctional services shown to reduce the risk of reoffending (Aharoni et al., 2020). The unspecified (control) condition did not state any information about the costs or benefits of incarceration.^1^

To accommodate site-specific constraints, data could not be collected from all sites simultaneously, and the likely response rate from each site was not estimable. For these reasons, we devised an adaptive assignment procedure to maximize power for the comparison of greatest theoretical interest to us: cost vs. unspecified. In this procedure, the first 110 participants (cohort 1) were pseudo-randomly assigned (with equal probability, using the Qualtrics assignment algorithm) to cost or unspecified, the next 55 participants were assigned to the benefit condition (cohort 2), and all subsequent participants were pseudo-randomly assigned to any one of the three conditions (cohort 3). This strategy was successful in meeting our sample size targets for each condition in order of priority. A one-way Analysis of Variance did not reveal any differences in the dependent measures between cohorts, either for the subjectivized sentencing score (*p*=0.972) or the objective sentencing score (*p*=0.326). Likewise, survey completion date was not correlated with punishment scores, either for the subjectivized scale (*r*=0.01, *p*=0.89) or the objective scale (*r*=0.07, *p*=0.37).

### Materials and Procedures

Prosecutors were surveyed electronically *via* the Internet between January–May, 2021. Survey invitations with standardized instructions were distributed electronically to the organization’s roster of active prosecutors *via* direct email communication or newsletter. For cohorts 1 and 2, participants were informed that, for each submission, a small monetary contribution ($5.00) would be donated to a preapproved service organization of the participant’s choice (*The National Offender Re-entry Association*, *The National Crime Victim Law Institute*, or *The National Association for Court Management*). Cohort 3 received no such opportunity so as to comply with their internal policy requirements. All responses were anonymous. After providing their consent, the survey instructions were presented as follows:

In this task, imagine you are a prosecutor for a fictitious jurisdiction in the U.S. You will read a case summary about an adult defendant who has been found guilty of a level 3 drug felony (Distribution of Controlled Substances), for which the statutory punishment range is 0–10years. The judge is likely to sentence the defendant to prison. As the prosecutor on this case, you may now make a recommendation to the judge regarding how long the defendant should be sentenced, if at all. Then, you will be asked questions about yourself and about the case, so please read very attentively.

The criminal case summary, adapted from Rachlinski et al. (2013) described a fictitious defendant convicted of drug trafficking, kept brief to respect participants’ time and minimize statistical noise. The full text stated:

Joseph Campbell, an unemployed male, was arrested at a party for selling 80 grams of methamphetamine.^2^ Joseph was found guilty of Distribution of Controlled Substances. The evidence at trial, which included testimony from an undercover police officer and two other witnesses, showed convincingly that he exchanged the methamphetamine for $8,000 in cash. Joseph is 30-years-old, has a spotty employment record, and has a history of drug addiction. He has 2 prior convictions for the sale of methamphetamine, for which he completed probation.

As a validation of the case summary, the vast majority of our sample (97.2%) agreed with the statement that there was enough evidence to support the defendant’s conviction. Following the case summary, the Cost and Benefit conditions included this additional statement:

Incarcerating Joseph will (cost/save) the county $30,000 to $35,000 per year in the form of (direct expenses/reduced crime). This is money that (could otherwise have been/can instead be) spent on reentry support services shown to reduce the risk of reoffending.

To adjust for jurisdiction-level variation in sentencing ranges, participants first provided a subjectivized sentencing judgment on an ordinal scale from (−4) “minimum allowable” to (+4) “maximum allowable.” Then participants made an objective sentencing recommendation using a ratio scale ranging from 0–10 years in prison, roughly commensurate with many state sentencing schemes. The dependent measures stated: “Based solely on these facts, how much should the defendant be punished for this offense?,” followed by “Based solely on these facts, how much time in prison should the defendant receive for this offense?” Sentencing scores were assessed for their association with standard demographic variables to help evaluate the independence of any observed effects of our manipulations. After providing a sentencing recommendation, they were asked to briefly justify, using text entry, how they arrived at that recommendation (i.e., “What considerations most strongly influenced your decision?”). These qualitative responses were coded by a trained rater who was blind to the study hypotheses. The rater coded the presence or absence of reasons supporting or opposing a given sentencing (i.e., potential aggravating and mitigating factors). For the opposing reasons, the rater also coded whether or not the response specifically referred to sentencing costs as a reason for mitigation.

Participants then answered a series of manipulation checks, credibility checks, and attention checks, including multiple choice questions on what crime the defendant was convicted of, whether they believed there was enough evidence to support his conviction, how much money the fictitious county would ostensibly spend to incarcerate or save by incarcerating the defendant, and whether this monetary sum was more or less than the participant expected. To search for possible discrepancies between participants’ explicit attitudes and their sentencing behavior, they were asked how much they disagree or agree with the statement that judges should consider the monetary costs of the sentence before deciding how much an offender should be sentenced. They were also asked how costly the defendant’s term of incarceration would need to be to persuade them to reduce their sentence recommendation. In recognition that prosecutors’ punishment recommendations could be influenced by other variables not central to our hypothesis, we collected self-report information on participants’ jurisdiction, unit, level of government, and years of experience for purposes of experimental control. However, jurisdiction and unit responses lacked sufficient power to justify analysis. Participants were asked standard demographic questions including age, gender, and political ideology. Last, cohorts 1 and 2 were given an opportunity to select one of three pre-approved non-profit service organizations to which the investigator would donate ($5.00) on their behalf, or they could indicate no preference. The survey did not allow participants to change their answers once submitted. Median survey completion time was 8.7min. Study data were analyzed using IBM SPSS v. 26. All study procedures were performed in accordance with the regulations of Georgia State University’s Institutional Review Board and conditioned on informed consent.

## Results

Both hypotheses were fully supported. We found a main effect of information salience on subjectivized sentencing recommendation, based on a rating scale from minimum to maximum allowable, *F*(2, 175)=3.37, *p*=0.037 (One-way ANOVA). Planned comparisons revealed that punishment recommendations were significantly higher when the cost of incarceration was not specified (*M*=0.12, *SE*=0.24, 95% CI [−0.36, 0.60]) than when it was specified (*M*=−0.81, *SE*=0.27, 95% CI [−1.34, −0.29], *p*=0.010; Fisher’s LSD). In contrast, consistent with our hypothesis, subjectivized punishment recommendations in the benefits condition (*M*=−0.25, *SE*=0.31, 95% CI [−0.86, 0.36]) did not statistically differ from the unspecified condition, *p*=0.169.

The same test was applied to participants’ objective sentencing recommendations (from zero to 10years in prison). Once again, the predicted main effect was found, *F*(2, 175)=3.31, *p*=0.039. Here, sentencing recommendations were 31.3% higher when the cost of incarceration was not specified (*M*=3.69, *SE*=0.30, 95% CI [3.10, 4.28]) than when it was present (*M*=2.81, *SE*=0.32, 95% CI [2.17, 3.45], *p*=0.047). Sentences in the cost condition were also lower than those in the benefit condition (*M*=4.00, *SE*=0.38, 95% CI [3.25, 4.74], *p*=0.018). However, as predicted, sentences in the benefit and unspecified conditions did not differ, *p*=0.528 (See Figure 1).

**Figure 1:**
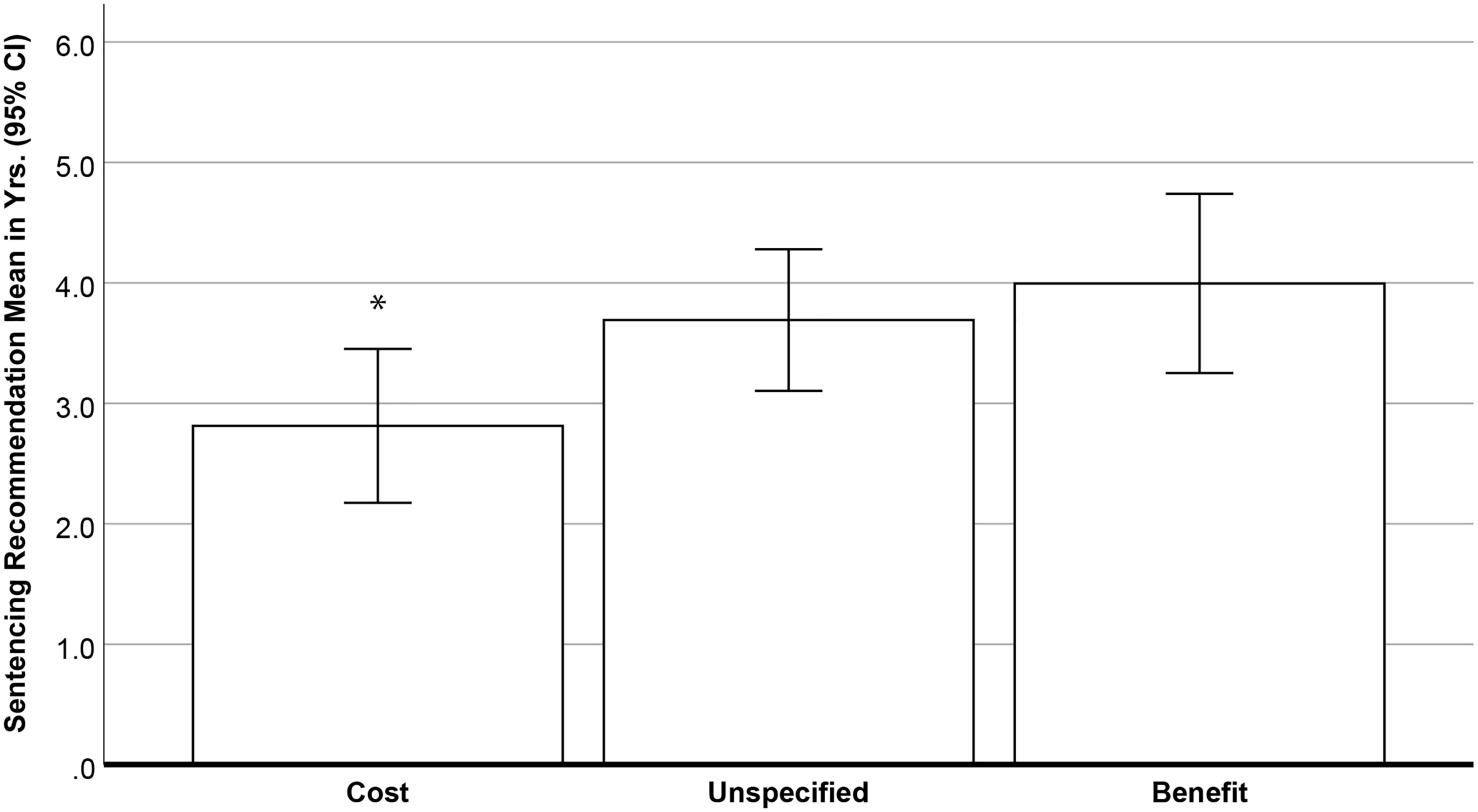
Mean objective sentencing recommendations when information about the costs (*n*=61) or benefits (*n*=45) of incarceration was present or unspecified (*n*=72). Cost<Unspecified^*^; Cost<Benefit^*^; ^*^p<0.05.

Some of our sample’s demographic characteristics deviated from norms among U.S. prosecutors. For example, the proportion of female prosecutors in our sample was more than twice the national average of 24% (Women Donors Network, 2019). Therefore, to examine the possibility that the observed cost salience effect could be explained by demographic anomalies, we conducted a series of tests (chi-square and one-way ANOVA) to search for relationships with known demographic characteristics. As expected, the distributions of gender, *χ*^2^(2)=0.24, *p*=0.30, level of government, *χ*^2^(2)=2.17 *p*=0.34, political ideology, *F*(2, 159)=0.86, *p*=0.43, and years of prosecutorial experience, *F*(2, 164)=1.07, *p*=0.34, did not differ across experimental conditions, disqualifying them from the conditions necessary for confounding (see Jager et al., 2008). Participant age was not uniformly distributed across condition: those in the benefit condition (*M*=38.29) were somewhat younger than those in the cost condition (*M*=43.36) and absent condition (*M*=43.77), *F*(2, 152)=5.229, *p*=0.006. However, age was not correlated with punishment, once again eliminating concerns about confounding influence (Jager et al., 2008; See Table 1 for Pearson correlations; See Supplementary Tables S1 and S2 for breakdown of demographic variables by condition.).

**Table 1:** Pearson correlation coefficients (*r*) between dependent measures and standard demographic variables.

		Sentencing Recommendation	Gender	Age	Years Experience	Political Ideology
Subjectivized Punishment	*r*	0.790^***^	0.018	0.126	0.223^**^	0.344^**^
	value of *p*	0.000	0.815	0.118	0.004	0.000
	*n*	178	164	155	167	178
Sentencing Recommendation	*r*		0.074	0.091	0.221^**^	0.359
	value of *p*		0.343	0.263	0.004	0.000
	*n*		164	155	167	162
Gender (*f*=0; *m*=1)	*r*			0.053	0.094	−0.021
	value of *p*			0.520	0.237	0.792
	*n*			151	161	156
Age	*r*				0.752^***^	0.136
	value of *p*				0.000	0.096
	*n*				155	150
Years Experience	*r*					0.169^*^
	value of *p*					0.033
	*n*					159

* *p*<0.05;

** *p*<0.01;

*** *p*<0.001.

Overall, our sample disagreed with the statement that judges should consider the monetary costs of their sentences, *t*(172)=−12.09, *p*<0.001, *M*=1.44, *SE*=0.12, with 74.6% expressing disagreement, 16.8% expressing agreement, and 8.7% expressing no opinion. But critically, when bracketing the subset of participants who did not agree with the statement, the mitigating effect of cost exposure on punishment judgments still persisted even in this disapproving subgroup; this was the case both for the subjectivized sentencing recommendation, *F*(2, 141)=4.65, *p*=0.011, and the objective sentencing recommendation, *F*(2, 141)=3.42, *p*=0.035 (one-way ANOVA), suggesting an inconsistency between prosecutors’ stated preferences (to ignore costs) and their revealed preferences (to value those costs).

This dissociation between stated and revealed preferences also extended to participants’ qualitative justifications for their sentences. When asked to justify the sentencing recommendation that they provided, 85.4% of the sample posited at least one reason to incarcerate, such as dangerousness or deservingness. However, participants in the benefit condition were no more likely to cite such positive factors than those in the other two conditions, *χ*^2^(2)=3.63, *p*=0.16. By contrast, just 4.5% of the sample cited sentencing costs as a factor in their recommendations. Despite the fact that cost exposure mitigated participants’ sentencing recommendations, these participants were no more likely than those in the other conditions to explicitly cite the costs as a sentencing factor, *χ*^2^(2)=3.57, *p*=0.17 (chi-square test). This pattern suggests that the predicted effect of cost exposure on sentencing recommendations may operate on an implicit rather than explicit level.

As an incentive to participate in this study, most (93.3%) of the participants exercised an opportunity to donate a small sum ($5.00 ea.) to a pre-approved criminal justice service organization of their choice. On average, the majority (70.7%) chose to donate to a victim services organization instead of an offender services organization (28.0%) or to court administration (1.3%). However, participants who were exposed to information about the costs of incarceration were more than twice as likely to donate to the offender service organization (39.6%) than those who received no fiscal information (18.6%), *χ*^2^(2)=6.05, *p*=0.048 (chi-square test). This result shows that incidental exposure to sentencing cost information increases investment in offender welfare. Critically, this change in donation behavior demonstrates an effect of our manipulation on prosecutors’ real-world choices (See Supplementary Material for additional analyses conducted.)

## Discussion

This study was designed to test the impact of cost-benefit salience on sentencing recommendations made by prosecutors. Since prosecutors are adversaries to the defendant and are not required to answer for the costs of their sentences, one might not expect them to place much value on the costs of their sentences. And indeed, when cost information was hidden, they did not. As predicted, prosecutors rendered prison sentence recommendations that were substantially (over 30%) longer when the decision cost information was absent than when it was present. Conversely, punishments in the unspecified control condition were as high as those under exposure to fiscally equivalent benefit information, suggesting a sentencing strategy that incorporates benefits of incarceration by default, but not costs. These effects could not be explained by known demographic variables. This asymmetry in how prosecutors weigh the benefits vs. costs of incarceration under the status quo appears to shift sentencing judgments upward relative to more informationally transparent contexts, reflecting the concerns of the so-called correctional free lunch (Zimring and Hawkins, 1991, 211–215). However, strikingly, direct exposure to relatively limited information about sentencing costs is sufficient to elicit their consideration, on balance with the benefits, reducing recommended prison terms substantially.

Our study found distinct discrepancies between prosecutors’ overt attitudes and their sentencing behavior, raising questions about which of these better represents their underlying motivations. The fact that cost exposure changed their donation behavior forces us to also take their sentencing behavior seriously. But the fact that most participants expressly opposed the use of cost information by judges seems incongruent with that sentencing behavior. This incongruity was more pronounced than in studies of laypeople, who, as a whole, did not strongly oppose (or support) the use of cost information by judges (Aharoni et al., 2018, 2019). The incongruity between prosecutors’ self-reported attitudes and sentencing judgments suggests that the selective effect of cost exposure on sentencing judgments may be somewhat unreflective, operating on an implicit level. Such an interpretation is consistent with the operation of a heuristic reasoning process, wherein people tend to place greater consideration on factors that are most readily available (Tversky and Kahneman, 1973) while neglecting others (McCaffery and Baron, 2006). For prosecutors, costs that are out of sight seem to be out of mind.

In practice, prosecutors overwhelmingly are not required to consult information about the expected costs of their sentencing recommendations. Indeed, deontological (duty based) legal theory prescribes that sentencing judgments should be made on strictly retributive grounds, without regard for the punishment’s potential consequences (Packer, 1968). But as an empirical matter, this study suggests that prosecutors do recognize inherent tradeoffs between time incarcerated and other valued opportunities (e.g., reentry services), consistent with a consequentialist (utility based) strategy and with research on judges and laypeople (Baron and Leshner, 2000; McCaffery and Baron, 2006; Bartels and Medin, 2007; Rachlinski et al., 2013; Twardawski et al., 2020). What is surprising is that they only appear to engage in this consequentialist accounting when the costs are made salient.

Studies have shown that joint evaluation of multiple perspectives improves decision consistency in moral dilemma judgments (Barak-Corren et al., 2018) and criminal sentencing judgments (Aharoni et al., 2020). It is thus likely that simultaneous exposure to both the costs and benefits of incarceration will improve sentencing consistency, perhaps by motivating decision makers to pursue a net-optimal sentence rather than a purely strategic, adversarial one (Miller, 2019). This could be achieved by systematically consulting sentencing costs alongside the benefits while prosecutors prepare their presentence reports (Allyn, 2018; Alpert, 2021).

This study raises several questions for future research on punishment cost-benefit salience. First, although our sample was regionally diverse, it is not necessarily generalizable to U.S. prosecutors as a whole. For example, our sample had a greater representation of women and leaned slightly liberal on our self-report scale. Furthermore, it was not possible to control for broader contextual factors, such as the potential influence of election campaigns, pandemic-related policy changes, or other time-locked covariates (Berdejó and Yuchtman, 2013). Tests of temporal covariation, gender, political ideology, and other common variables did not yield evidence of confounding, but other variables (e.g., race) were not collected. Future research can more fully evaluate and control for possible third variables that can threaten sample representativeness by employing a fully randomized or propensity-weighted assignment procedure that captures additional demographic and contextual factors. Second, the crime scenario was selected to be moderate in seriousness. Evidence from research using laypeople suggests that mitigating effects of cost exposure may also operate among more serious crimes such as aggravated robbery and home invasion (Aharoni et al., 2018), but additional research is needed to test this hypothesis in legal practitioners as well with capital offenses. Third, there is a necessary tradeoff between a study’s degree of experimental control and its ecological validity (Merrall et al., 2010). We used a hypothetical case narrative and restricted participants’ sentencing options in order to demonstrate a causal effect of information salience on sentencing behavior. Relatedly, there are inherent difficulties in quantifying the true costs and benefits of incarceration. We matched the costs and benefits numerically in order to permit an unbiased comparison of their relative influence. Future research should probe salience effects using more complex, realistic manipulations and measures, including direct measurement of belief in the manipulated information, or using archival legal data to compare sentencing outcomes before and after enactment of cost transparency policies. However, the fact that cost exposure in our study precipitated greater allocation of charitable donations to an offender service organization indicates that our hypothetical manipulation did exert real-world influence.

Despite these limitations, the present findings demonstrate that, lacking immediate exposure to sentencing decision costs, prosecutors may favor substantially harsher sentences than they would under more transparent conditions. Since longer sentences increase custodial incidence rates, this selective neglect of sentencing costs is likely to be a systemic, but previously unrecognized contributor to mass incarceration. This effect could apparently be reversed by making sentencing costs as transparent as the benefits. Policymakers should consider such evidence when designing the choice architecture that supports such impactful decisions. However, the question is not whether shorter sentences are necessarily better. It is whether legal authorities who represent the public interest can be expected to make sound legal judgments when one half of the relevant information (in this case, cost information) is consistently censored. This study thus makes a critical contribution to current policy debates on mass incarceration and empowers legal practitioners and their electorates to take seriously the role of information transparency in criminal sentencing.

## Data Availability Statement

The original contributions presented in the study are publicly available. This data can be found at https://osf.io/u749f.

## Ethics Statement

The studies involving human participants were reviewed and approved by Institutional Review Board, Georgia State University. The participants provided their written informed consent to participate in this study.

## Author Contributions

EA, HK-O, and SB conceived the project and developed the empirical approach. EA performed the data collection and analysis and wrote the first draft of the manuscript. All authors contributed to discussions about the paper’s focus, proposed edits, and approved the final version of the manuscript for submission.

## Funding

This project received funding from the nonprofit Charles Koch Foundation. The funders had no role in study conceptualization, design, data collection, analysis, decision to publish, or preparation of the manuscript.

## Conflict of Interest

The authors declare that the research was conducted in the absence of any commercial or financial relationships that could be construed as a potential conflict of interest.

## Publisher’s Note

All claims expressed in this article are solely those of the authors and do not necessarily represent those of their affiliated organizations, or those of the publisher, the editors and the reviewers. Any product that may be evaluated in this article, or claim that may be made by its manufacturer, is not guaranteed or endorsed by the publisher.

## Footnotes

^1^Our study design and materials were adapted from unpublished pilot research that produced similar results among university 146 undergraduates and 158 law students (See Supplementary Material for details).

^2^The mass of methamphetamine (80 grams) was strategically selected on the basis of personal communication with several legal practitioners who were not study participants. This amount was determined to be large enough to justify imprisonment while small enough to justify prosecution at the county level in many jurisdictions.
