# Supplementary material for: Correctional “Free Lunch”? Cost Neglect Increases Punishment in Prosecutors
Source: Front Psychol. 2021 Nov 12;12:778293. doi: 10.3389/fpsyg.2021.778293 (PMC8633388; doi:10.3389/fpsyg.2021.778293)
Supplement: Supplementary file 1 [file Data_Sheet_1.docx]

**Supplementary Material**

1. **Prosecutor study: extended analysis**

One possible explanation for the effect of cost exposure on punishment judgments could be that prosecutors are rationally responsive to apparent changes in the decision’s utility. If the stated cost of incarceration was greater than what prosecutors expected, a consequentialist strategy (based on sensitivity to changes in utility) should produce punishment recommendations that are smaller than those without that cost information. This was tested by assessing whether participants’ expectations tracked their punishment judgments. In response to a question about whether the monetary amount was less than (-1), about the same as (0), or more than participants expected (+1), participants in the cost condition reported that it was slightly more than they expected, *t*(60) = 2.62, *p* = .011, *M* = 0.21, *SE* = 0.08 (one-sample *t*-test). However, if people’s judgments are in fact rationally responsive to the consequences, then expectations about the decision’s benefits should also influence punishment judgments. They did not. Participants in the benefit condition reported that the stated benefit of incarceration was slightly greater than they expected, *t*(44) = 12.71, *p* < .001, *M* = 0.87, *SE* = 0.068, and yet punishment scores in this condition did not differ from the unspecified condition containing no cost or benefit information. If the null effect of benefit exposure on punishment were simply due to disbelief in the manipulation, then this problem of face validity should also have nullified the effect of cost exposure on punishment, but it did not. Thus, the asymmetrical effect of cost-benefit exposure on punishment judgments is not easily explained by rational consideration of new information or by variation in face validity.

We tested whether prosecutors at some levels of government (local, state) might be more responsive to the observed effect of cost exposure than others. However, the level of government did not exert a main effect on subjectivized sentencing recommendations, *F*(1, 160) = 0.01, *p* = .91, nor did it interact with cost, *F*(1, 160) = 0.66, *p* = .42 (two-way ANOVA). Cost was defined as present vs. absent, where the absent category comprised a combination of the unspecified and benefit conditions to improve statistical power for this test. The same null pattern was found for the objective sentencing measure with respect to the main effect of government level, *F*(1, 160) = 1.82, *p* = .18, and its interaction with cost, *F*(1, 160) = 0.06, *p* = .82.

Participants were asked how expensive incarceration would need to be to convince them to reduce their sentencing recommendation. On the whole, they expressed that the annual cost of incarceration would need to be at least $100K (MD = $100K - $300K) to consider reducing their sentences, suggesting that they are willing to tolerate relatively high costs to achieve their punishment goals. Indeed, the modal response was that “no cost would be large enough” (39.9%). So the fact that punishment judgments were predictably responsive to a cost manipulation below this median threshold (i.e., $30,000 to $35,000 per year) again suggests an inconsistency between prosecutors’ stated preferences and revealed preferences.

We tested whether the observed effect of cost exposure on sentencing recommendations depended on political ideology. Although the overall linear regression model was significant, *R*^2^ = .147, *p* < .001. There was a main effect of political ideology (-3 = very liberal; +3 = very conservative) wherein self-reported conservatism was positively associated with sentencing length, B = .495, *p* < .001, but the interactive effect of political ideology and cost (-1 = present; +1 = absent) on subjectivized sentencing score was not significant, *p* = .717 (the absent category was comprised of a combination of the unspecified and benefit conditions to improve statistical power for this test). The same pattern held for an equivalent model using the objective sentencing score as the dependent measure. The model was significant, *R*^2^ = .155, *p* < .001, and there was a similar main effect of political ideology, B = .699, *p* < .001, but this variable did not interact with cost exposure, *p* = .669.

Cost remained a significant predictor of punishment when controlling for years of experience as a prosecutor, *F*(2, 163) = 5.08, *p* = .007. Specifically, punishment scores were significantly lower in the cost condition (*M* = -1.06, *SE* = 0.26, 95% CI [-1.58, 0.54]) than the unspecified condition (*M* = 0.06, *SE* = 0.24, 95% CI [0.42, 0.54]). The same pattern was observed for sentencing recommendations, *F*(2, 163) = 6.04, *p* = .003. Sentences were significantly shorter in the cost condition (*M* = 2.45, *SE* = 0.31, 95% CI [1.84, 3.07]) than the unspecified condition (*M* = 3.61, *SE* = 0.29, 95% CI [3.04, 4.18]).

Table S1. Number of participants for each demographic variable by experimental condition

|  | **Cost** | **Benefit** | **Absent** | **Total** |
| --- | --- | --- | --- | --- |
| Females | 39 | 27 | 41 | 107 |
| Males | 19 | 21 | 25 | 65 |
| Gender other or not specified | 3 | 2 | 2 | 5 |
| Local gov’t | 49 | 39 | 49 | 137 |
| State gov’t | 10 | 9 | 16 | 35 |
| Gov’t other or not specified | 2 | 3 | 2 | 7 |

Table S2. Raw Mean (SD) of each demographic variable by experimental condition

|  | **Cost** | **Benefit** | **Absent** | **Total** |
| --- | --- | --- | --- | --- |
| Political Ideology | -0.95 (1.08) | -0.92 (1.38) | -0.66 (1.51) | -0.83 (1.34) |
| Prosecutorial Experience | 11.00 (7.32) | 9.98 (6.84) | 12.07 (7.67) | 11.13 (7.33) |
| Age | 43.36 (9.47) | 38.29 (8.12) | 43.77 (9.79) | 42.11 (9.50) |

1. **Pilot study of undergraduate students**

The design of the pilot study of undergraduates (N = 146) was identical to that of the prosecutor study.

The overall effect of our manipulation on sentence length was significant, *F*(2, 143) = 5.23, *p* = .006 (one-way ANOVA). Consistent with our hypothesis, planned comparisons using Fisher’s LSD revealed that sentences were significantly (~ 33%) lower in the presence of cost information (*M* = 3.13, 95% CI [2.35, 3.91]) relative benefit information (*M* = 4.69, 95% CI [3.95, 5.43], *p* = .005), or when cost-benefit information was unspecified (*M* = 4.62, 95% CI [3.91, 5.33], *p* = .006). However, sentences in the benefit condition and unspecified condition did not differ from each other, *p* = .885.

In response to a question about whether the monetary amount was less than, about the same as, or more than participants expected, participants in the cost condition reported that it was more than they expected, *t*(43) = 5.70, *p* < .001, *M* = 0.57, *SE* = 0.10 (one-sample *t*-test). Participants in the benefit condition also reported that the stated benefit of incarceration was greater than they expected, *t*(47) = 7.54, *p* < .001, *M* = 0.71, *SE* = 0.09, and yet punishment scores in this condition did not differ from the unspecified condition containing no cost or benefit information.

One possible interpretation is that the null effect of benefit information on punishments reflects mere disbelief in the manipulated information. After all, the stated value of the benefit apparently exceeded participants expectations. But if disbelief in the manipulation were the true cause of the null effect, it fails to explain how the effect of equivalent cost information on punishment persisted despite that information also exceeding participants expectations. Moreover, because undergraduates presumably have less subject-matter expertise than prosecutors about sentencing cost-benefit information, yet they still exhibit a selective effect of cost exposure on sentencing recommendations, this further alleviates concerns about the face validity of the manipulations.

1. **Pilot study of law students: results**

The design of the pilot study of law students (N = 158) was identical to that reported in Rachlinski and colleagues (2013). Participants were randomly assigned to one of three cost conditions: True cost ($32K per inmate per year, Low cost ($16K per inmate per year), or Unspecified cost. There was no benefit condition in this survey, and we did not collect information about whether the cost information presented differed from participants’ expectations.

The overall effect of cost type on sentence length was marginally significant, *F*(2, 155) = 2.43, *p* = .091 (one-way ANOVA). Consistent with our primary hypothesis, planned comparisons using Fisher’s LSD revealed that sentences were significantly lower in the True cost (*M* = 2.81, *SE* = 0.27, 95% CI [2.27, 3.35]) condition relative to the Unspecified cost (*M* = 3.62, *SE* = 0.29, 95% CI [3.04, 4.19]) condition, *p* = .046. However, sentences in the Low cost condition (*M* = 2.87, SE = 0.29, 95% CI [2.30, 3.44]) did not differ from those in the True cost condition, *p* = .884. Instead, they were marginally lower than those in the Unspecified condition, *p* = .071, suggesting that even artificially low costs might be sufficient to motivate restraint.
